# Genetic and epigenetic changes in host *ABCB1* influences malaria susceptibility to *Plasmodium falciparum*

**DOI:** 10.1371/journal.pone.0175702

**Published:** 2017-04-19

**Authors:** Himanshu Gupta, Sima Chaudhari, Ayushi Rai, Smitha Bhat, Pratima K Sahu, Manjunath H. Hande, Sydney C. D’Souza, Umakanth Shashikiran, Kapaettu Satyamoorthy

**Affiliations:** 1 Department of Biotechnology, School of Life Sciences, Manipal University, Manipal, Karnataka, India; 2 Department of Biochemistry & Molecular Biology, VIMSAR, Burla, Sambalpur, Odisha, India; 3 Department of Medicine, Kasturba Medical College, Manipal, Manipal University, Karnataka, India; 4 Department of Medicine, Kasturba Medical College, Mangalore, Manipal University, Karnataka, India; 5 Department of Medicine, Dr TMA Pai Hospital, Udupi, Karnataka, India; Institut national de la santé et de la recherche médicale—Institut Cochin, FRANCE

## Abstract

Multiple mechanisms such as genetic and epigenetic variations within a key gene may play a role in malarial susceptibility and response to anti-malarial drugs in the population. *ABCB1* is one of the well-studied membrane transporter genes that code for the P-glycoprotein (an efflux protein) and whose effect on malaria disease predisposition and susceptibility to drugs remains to be understood. We studied the association of single nucleotide variations in human *ABCB1* that influences its function in subjects with uncomplicated and complicated malaria caused by *Plasmodium falciparum (Pf)*. Global DNA methylation and *ABCB1* DNA promoter methylation levels were performed along with transcriptional response and protein expression in subjects with malaria and healthy controls. The rs2032582 locus was significantly associated with complicated and combined malaria groups when compared to controls (*p* < 0.05). Significant DNA methylation difference was noticed between case and control (*p* < 0.05). In addition, global DNA methylation levels of the host DNA were inversely proportional to parasitemia in individuals with *Pf* infection. Our study also revealed the correlation between *ABCB1* DNA promoter methylation with rs1128503 and rs2032582 polymorphisms in malaria and was related to increased expression of ABCB1 protein levels in complicated malaria group (*p* < 0.05) when compared to uncomplicated malaria and control groups. The study provides evidence for multiple mechanisms that may regulate the role of host *ABCB1* function to mediate aetiology of malaria susceptibility, prognosis and drug response. These may have clinical implications and therapeutic application for various malarial conditions.

## Introduction

Malaria is the world’s most common yet serious public health problem accounted globally for 214 million diagnosed cases and 438,000 deaths during 2015 [1]. The invasion of *Plasmodium falciparum* (*Pf*) into erythrocytes leads to the development of clinical features of malaria such as impaired consciousness, hyperpyrexia, hyperbilirubinemia, hyperparasitemia, severe anemia, cerebral malaria, renal failure, acute respiratory distress syndrome, hypoglycemia, acidosis and hemoglobinuria [2–4]. The molecular basis for the pathogenesis of malaria, prognosis and anti-malarial drug response remain to be completely understood [5]. However, recent studies have begun to reveal the inter-individual differences in malaria susceptibility and drug response as a result of genetic variation within the population [6–10]. Similarly, levels of global and gene specific methylation changes in cytosine residues and in DNA methylation variant such as 5-hydroxymethylcytosine along with other epigenetic changes such as in small RNAs may also drive population specific differences to disease susceptibility and its progression to defined clinical conditions [11–16]. These epigenetic alterations are profoundly influenced by diet, race, age, sex and various other environmental factors which can have an impact on the overall phenotypic characteristics [17, 18].

*ABCB1* gene belongs to ABC transporter gene family B and codes for P glycoprotein (P-gp) [19]. P-gp, also called as multidrug resistance protein 1 (MDR1), is usually expressed in intestine, kidney, liver, blood brain barrier, spinal cord, testes and placenta, and acts to guard efflux of xenobiotics as well as endogenous toxic substances from these tissues [18,20]. The expression of ABC transporters during infection in erythrocytes and other cells is of special interest to understand the molecular basis of malaria, as altered multidrug transporter expression could modulate the effect of antimalarial drugs to the intracellular parasite [21] and toxic effect to the host. In addition, studies have revealed that the presence of threshold quantity of malaria parasite in patients induces the synthesis of inflammatory cytokines such as TNF- α, IL-1 and IL-6 as a first line defence mechanism [22–24]. Even though reduced P-gp mRNA and protein expression was observed during active inflammation in colonic epithelium in ulcerative colitis patients, these pro-inflammatory cytokines have time dependent and dose dependent effect on P-gp expression [25, 26]. The pro-inflammatory cytokines are also involved in pathways that can enhance expression of *ABCB1* gene [27] thus regulating the efflux mechanisms to protect the host against deleterious mechanisms of infection. At the level of *ABCB1* gene promoter, increased expression of P-gp and its function has been suggested due to epigenetic changes induced by the cytokines [27]. It is also reported that toxic by-products of hemoglobin degradation may also lead to induction of *ABCB1* gene due to global DNA hypomethylation [28]. In the context of model systems and other diseases, hyperbilirubinemia is reported to be involved in overexpression of *ABCB1* gene [29, 30]. Apart from epigenetic, the substrate specificity, efflux mechanisms, expression and mRNA stability due to single nucleotide polymorphisms (SNPs) in human *ABCB1* [31] may also play a role in determining susceptibility to malaria.

*ABCB1* gene is highly polymorphic and more than 50 variants (SNPs) has been reported in the coding region among which rs1128503, rs2032582 and rs1045642 are the most common and these have been associated with various diseases including cancer, epilepsy, respiratory diseases, malaria, asthma, cardiovascular disease etc [19, 32–34]. However, there have been conflicting reports on the status and validity of *ABCB1* SNP associations with drug resistance and response to treatment [35].

The ability of cytokines to induce epigenetic changes, as well as autoinduction of *P450* (*CYP*) and *ABCB1* gene by malarial parasite, degraded by-products, anti-malarial drug and its derivative, suggest that malarial severity, genetic and epigenetic status of *ABCB1* gene and effect of antimalarial drug might be interrelated [18, 26, 27, 36]. Hence in this study, we tested the hypothesis that the activation or inactivation of *ABCB1* gene due to genetic and epigenetic changes may be regulated by malaria infection in order to eliminate hemoglobin degradation products. Towards this, we tested for global and gene specific DNA methylation changes and association of SNPs in *ABCB1* gene in individuals with sub-types of *Pf* induced malaria.

## Material and methods

### Participant’s recruitment

Individuals with malaria visiting Kasturba Hospital, Manipal and Kasturba Medical College, Mangalore, India, were enrolled during the malaria season over a period of 3 years spanning 2011 to 2013. This study was approved by the Institutional Ethical Committee of Kasturba Hospital, Manipal. The inclusion and exclusion criteria of study participants are presented in S1 Table. All the participants were informed about the study in advance and signed written informed consents were collected. After obtaining prior informed consent, 5ml of venous blood was acquired from 100 complicated and 100 uncomplicated *Pf* infected (200 cases in total) and 200 matched control participants in ethylenediaminetetraacetic acid (EDTA) vacutainers and further used for extraction of genomic DNA. These patient samples were divided into complicated and uncomplicated malaria groups. Complicated malaria was characterized as per WHO 2010 guidelines and refers to the presence of one or more organ dysfunction due to malaria infection. Information on different variables inclusive of platelet count, total bilirubin, serum creatinine, hemoglobin, random glucose and G6PD levels along with age and sex ratio were collected as demographic data of participants which are as presented in Table 1. The presence of parasites was detected in the blood using methods described previously [37]. Control subjects (n = 200) were those who reported no history of malaria upon the question-based evaluation performed by participated physician. The statistical parameters of control subjects were summarized as the mean age of 35.96 ± 13.83 years and sex distribution (male = 79% and female = 21%). The participants (malaria and control) age range was between 5 to 65 years.

**Table 1:** Demographic data and the clinical profile of the malaria patients recruited as participants for the study.

**Clinical Parameter–Malaria infected participants**			
	Complicated malaria (n = 100)	Uncomplicated malaria (n = 100)	^*a*^*p*-value
Age	31.66 ± 16.26	34.31 ± 16.0	0.22
Sex Male: Female:	77% 23%	80% 20%	
Hemoglobin (g/dl) Male: Female:	9.69 ± 0.58 9.61 ± 0.74	11.93 ± 0.93 12.22 ± 1.01	<0.001 <0.001
Platelet count (/mm^3^)	87072 ± 39284.8	91389 ± 41165.63	0.64
Serum creatinine (mg/dl)	2.72 ± 0.51	0.91 ± 0.29	<0.001
Total bilirubin (mg/dl)	5.22 ± 2.2	0.75 ± 0.45	<0.001
Glucose (mg/dl)	74.68 ± 10.9	112.06 ± 15.1	<0.001
G6PD levels (U/g Hb)	13.18 ± 4.18	13.53 ± 4.27	0.39
Parasitaemia (/QBC field)	≥ 100 parasites	<100 parasites	

Mann Whitney test ^a^ p-value < 0.05 considered to be significant.

### Western blotting analysis

Proteins were extracted from leukocytes of individuals with complicated, uncomplicated malaria and healthy controls after cell lysis in RIPA buffer. The extracts were kept on the ice and sonicated using 2mm probe ultrasonic processor. Sonication was operated at 40 amplitude for 15 minutes with periodic on and off for 15 seconds each in order to achieve complete lysis of the cells. A Bradford assay kit (Sigma) was used for the estimation of the protein concentration of each sample. In brief, 30μg of protein corresponding to individual of three groups were resolved in 8% SDS-PAGE and transferred to a nitrocellulose membrane (Sigma) prior to blocking with 5% BSA and overnight incubation at 4°C with 1:1000 diluted mouse monoclonal anti-P glycoprotein antibody (Calbiochem, clone C219). After subsequent treatment with anti-mouse IgG-HRP (Cell Signaling (1:5000)) as a secondary antibody, visualization of protein was mediated using ECL reagent (GE Healthcare) and ImageQuant LAS 4000 (GE Healthcare) was used for imaging. Antibody against β-actin (Cell Signaling (1:5000) was used as loading control.

### Genomic DNA extraction

The standard salting out precipitation method as described by Miller *et al*., was used to extract genomic DNA from participant’s blood [38]. Further, the integrity of each isolated DNA sample was checked on 0.8% agarose (Sigma) gels prepared in 1×tris-borate-EDTA buffer (8.9 mM Tris, 8.9 mM Boric acid, 2 mM EDTA) and DNA concentration was estimated with aid of NanoDrop1000 spectrophotometer.

### SNPs, Polymerase chain reaction (PCR), RFLP and DNA sequencing

**Fig 1:**
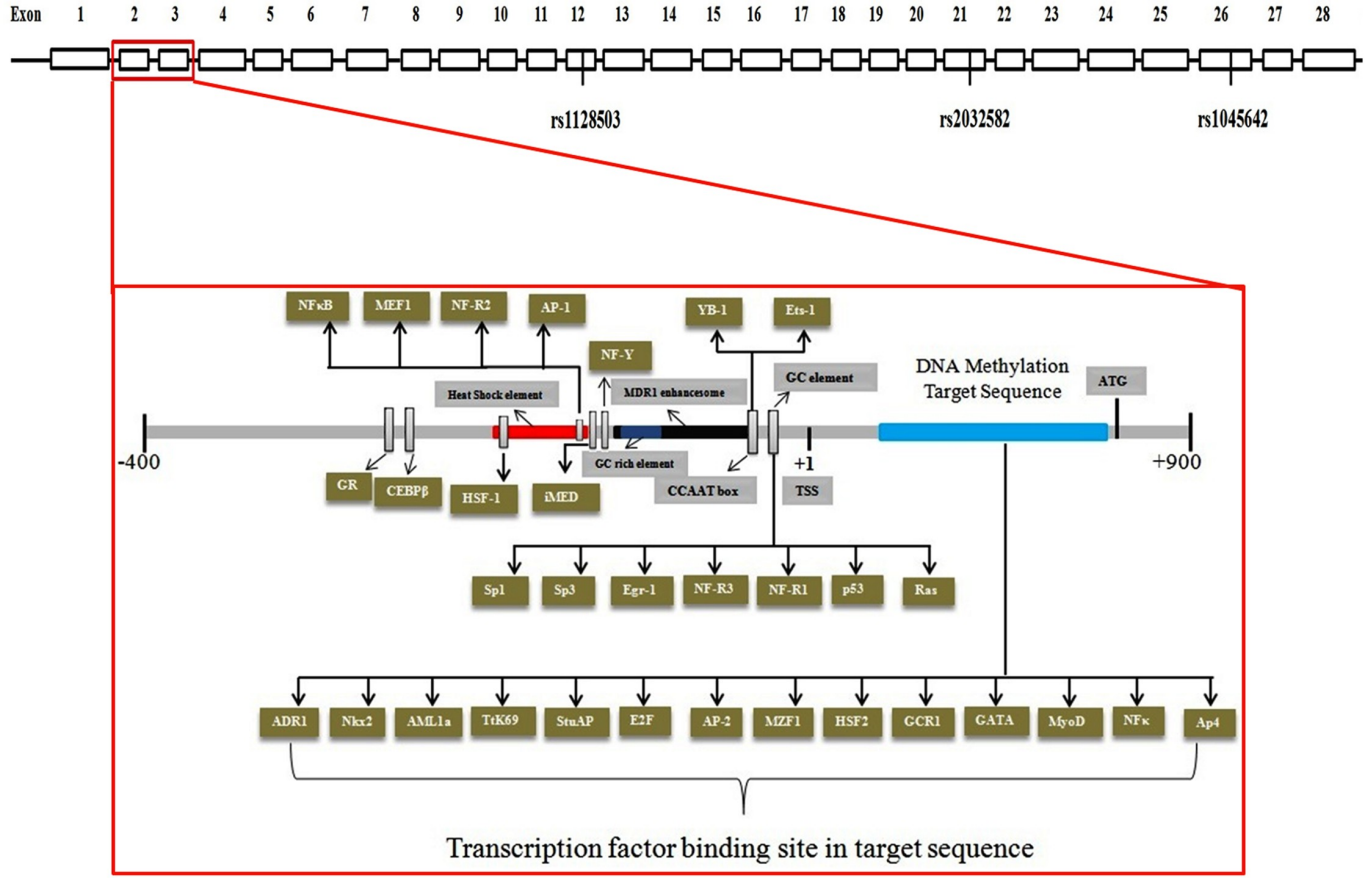
Schematic representation showing genome organization of *ABCB1* gene and the analyzed regions for SNPs and CpG sites (indicated in blue). Exons are indicated as boxes.

We analysed three key polymorphisms of *ABCB1* gene; rs1128503 in exon12 (NM_000927.3: c.1236C>T), rs2032582 in exon 21 (NM_000927.3: c.2677G>T/A) and rs1045642 in exon 26 (NM_000927.3: c.3435C>T) available on the chromosome 7q21.12 (Fig 1). The SNPs were selected based on data available in public domains such as dbSNP (www.ncbi.nlm.nih.gov/projects/SNP/) as well as the literature review on *ABCB1* variations, minor allele frequency and functional relevance. The online tool, Primer3 Input version 0.4.0 (http://frodo.wi.mit.edu/primer3/) was employed for designing primers (Forward primer: 5’-TATCCTGTGTCTGTGAATTGCC-3’; Reverse primer: 5’-CCTGACTCACCACACCAATG-3’) that selectively amplify the 370bp region including rs1128503 in exon12. Restriction Fragment Length Polymorphism (RFLP) was performed with restriction enzyme HaeIII. For the polymorphism in exon 21 (rs2032582), two PCR reactions were performed, with a common forward primer (5’ -TGCAGGCTATAGGTTCCAGG- 3’) and two different reverse primers (5’–GTTTGACTCACCTTCCCAG- 3’ and 5’ -TTTAGTTTGACTCACCTTCCCG- 3’) with a single base mismatch in the last reverse primer. PCR amplicons produced with the first of these primer pairs were 220bp long and had a BsrI restriction enzyme recognition site whereas, the second set with mismatch primer generates 224bp long PCR product with a BanI restriction enzyme recognition site G allele recognized in RFLP experiments. The polymorphism present in exon26 (rs1045642) was genotyped with restriction enzyme Sau3A1, after PCR amplification of 197bp sequence (forward primer: 5’–TGTTTTCAGCTGCTTGATGG- 3’; reverse primer: 5’–AAGGCATGTATGTTGGCCTC- 3’). Enzymes used were supplied by New England Biolabs (USA). Primer sequences were analysed for specificity and sequence homology using the nucleotide alignment search tool, BLASTn (http://www.ncbi.nlm.nih.gov/). Further optimization and determination of annealing temperature for the designed primer pairs were done on Veriti AB PCR 96 well thermal cycle machines. The PCR reaction was performed with the initial denaturation at 95°C for 5min, followed by 30 cycles of 95°C for 1min, 57°C for 1min, 72°C for 1min 30s, followed by final extension at 72°C for 10min, for all three polymorphisms except annealing temperature was 53°C for rs1128503. PCR products were visualized on 2% agarose (Sigma) gels in 1× TBE buffer. For validation of the PCR-RFLP results, sequencing of the PCR amplicons was performed for 30 randomly selected samples from each group of 3 SNPs. DNA sequencing was carried out by the modified dideoxy mediated chain termination method [39] using ABI Prism (Applied Biosystems, USA) 3130 genetic analyzer DNA sequencer and ABI Prism Big Dye Terminator v3.1 cycle sequencing kit. However, same primers designed for the PCR reaction were also employed for the sequencing reactions. The variations between the output sequencing result and reference sequence NG_011513 were analysed and identified by sequence alignment using NCBI blast.

### Bisulfite sequencing PCR

In order to validate the methylation status of 30 CpG sites within the *ABCB1* promoter in age-sex matched complicated malaria (n = 19), uncomplicated malaria (n = 21), all malaria (n = 40) and non-malaria (n = 40) participants, bisulfite sequencing was carried out on genomic DNA isolated from the blood. Bisulfite conversion of genomic DNA was carried out as per the manufacturer’s instructions for Zymo EZ Methylation Kit (Kit # D5002). We used one microgram of genomic DNA for optimum bisulfite conversion. After conversion, the DNA bound to the column was eluted in 20μl of sterile water. Converted DNA was amplified with the primer pair (forward 5’-GAAGTTTTTTGGTAAGTTTATGGG-3’ & reverse 5’-CTCATTAACCAAATACATAAACCTCA-3’) to selectively amplify 434bp region, which covers a downstream promoter region (+245 to +679) of *ABCB1* gene (Fig 1). The anticipation of CpG islands was done using the online tool MethPrimer using default parameters [40]. *NNAT* imprinted gene was picked as an experimental control, as demonstrated before *NNAT* gene is partially methylated in blood [41]. Converted DNA of malaria subjects and healthy individuals were amplified with the primer pair (forward 5’- ATTTATTAGGGTTTGGGGG-3’ & reverse 5’-ATCATCTACCCCATAAAACAAA-3’) to selectively amplify 424bp region (Chr 20: 35582248–35582671) of *NNAT* gene. The optimization and determination of the suitable annealing temperature of both primer pairs were achieve using Veriti AB PCR 96 well thermal cycle machines with a primer concentration of 10pmols. The PCR reaction was performed with the initial denaturation at 95°C for 5min, followed by 30 cycles of 95°C for 30sec, 58°C for 1min, 72°C for 1min 30s, followed by a final extension at 72°C for 10min. PCR products were checked on 1.5% agarose (Sigma) gels in 1× TBE buffer. Further, the sequencing of the purified PCR amplicons was carried out as described above. Sequence ABI files and methylation levels at a given CpG site were quantified using ESME software as previously described [42].

### Global DNA methylation analysis

Estimation of global methyl cytosine (mC) was performed by employing slightly modified Reverse-phase high performance liquid chromatography (RP-HPLC) method as described previously [43, 44]. Briefly, 1U of DNaseI enzyme (New England Biolabs, USA) was used to digest total of 1μg of human gDNA, denaturation was done at 100°C for 10min and immediately ice treatment was provided for few minutes, followed by the addition of 1U and 2U of Nuclease P1 (Sigma-Aldrich, Canada) and calf intestinal phosphate (New England Biolabs, USA) respectively. The content of 5-mC was assessed in duplicate by injection of the sample to the RP-HPLC using C18 columns (Grace Vydac, Hesperia, CA, USA). Isocratic mobile phase (a mixture of 50mM potassium dihydrogen phosphate (pH 3.5) and methanol in 9:1 ratio respectively) was delivered at the flow rate of 1ml/min. The percentage of 5-mC was determined by using the standard formula [(5-mC peak area) /(C peak area /5-mC peak area)] /100 (where C is Cytosine and 5-mC is 5-methyl Cytosine)].

### DNA Cloning of *ABCB1* promoter

The 949bp region of *ABCB1* promoter (-243 to +706) was amplified using primer pair (forward 5’-AATGTCCCCAATGATTCAGC-3’ & reverse 5’-CCATTCCGACCTGAAGAGAA-3’). PCR amplicon was first cloned into a pTZ57R/T vector (Thermo Fisher Scientific, USA). After the blue white screening, plasmids were isolated, restriction digested and direct DNA sequencing confirmed the clone. The PCR amplicon in pTZ57R/T vector was sub-cloned into a pGL3-Basic vector (Promega, USA) by using KpnI and HindIII restriction enzymes (New England Biolabs, UK) and was named as pGL3-ABCB1. The pTz57R/T plasmid was digested with NcoI and SmaI restriction enzymes (New England Biolabs, UK). Digested product (454bp) was sub-cloned into the corresponding restriction enzyme sites of ptk-luc vector and the clones were confirmed by DNA sequencing (ptk-luc-ABCB1). This 454bp region was also used for DNA methylation analysis by using bisulfite sequencing. The 949bp region contained two xenobiotics responsive element (XRE) sites at -51 to -47 and +233 to +237.

### Luciferase reporter assay

We checked the promoter activity of the PCR amplicon in HepG2 cell line. HepG2 cells were grown to approximately 80% confluence in T75 tissue culture flask. Transient HepG2 cell lines were produced by co-transfection of 1.0 x 10^5^ cells with 1 *μ*g each of test ptk-luc-ABCB1, pGL3-ABCB1, pGL3-basic vector, empty ptk-luc, pGL3-positive control vector and 50 ng of the pRL-SV40 vector (Promega, USA) as per the manufacture protocol of Lipofectamine LTX 2000 (Invitrogen, USA). 24 hours post transfection, the culture media was replaced with DMEM (HiMedia, Mumbai, India) supplemented with 10% fetal bovine serum (FBS) (HiMedia, Mumbai, India) containing 1 μM 3-methylcholanthrene (3MC) (positive control) and 1μM artemisinin with additional treatment for 24 h. Artemisinin and 3MC were obtained from Sigma-Aldrich (St. Louis, MO, USA) and dissolved in dimethyl sulfoxide (DMSO). Cell lysate collected 48 hours post transfection was used for estimation of luciferase (Firefly and Renilla) activities. The luciferase assay was performed with proDualGlo Luciferase Assay System (Promega, USA) in accordance with the manufacture’s instruction on a FB12 Single Tube Luminometer (Berthold, Germany). Three independent experiments were carried out for the assay, each experiment being performed in duplicates. For each experiment, ratios of firefly luciferase to Renilla luciferase readings were obtained and the average value of duplicates was further used for statistical analysis. The mean values of the test constructs were normalized to the activity of the pGL3-basic vector as well as empty ptk-luc whenever necessary that was promptly set at value 0 and 1 respectively and compared against pGL3-positive control vector. The outcomes of three independent experiments were presented as the mean ± standard deviation (SD).

### Statistical analysis

The free trial version of GraphPad InStat 3 program (GraphPad Software, San Diego, CA, USA) was used for data analysis. Control samples genotype frequencies were used to calculate Hardy Weinberg equilibrium. Genotype and allelic frequencies distributions were evaluated statistically with a χ^2^ test. At 95% confidence interval (CI), odds ratios (OR) were calculated and *p value* < 0.05 was considered as statistically significant. Clinical data of the two malaria groups were analysed with nonparametric Mann-Whitney test. Bonferroni method was applied so that corrected *p value* could be calculated. Bonferroni corrections for multiple SNPs were performed using the formula: α = 1- (1- α’)1/n (corrected for n comparisons, α’ = un-corrected *p value*, n = 3) [45]. Other statistical analysis such as haplotype and linkage disequilibrium (LD) analysis were performed using an online tool “SHEsis”. The software employs a partition-ligation-combination-subdivision expectation maximization algorithm for haplotype inference with multiallelic markers. Haplotype analysis was performed considering 0.03 as the lowest frequency threshold that ultimately eliminates all single haplotypes with a frequency below this value. Linkage disequilibrium of the three SNPs present within the *ABCB1* gene sequence was figured and graphically presented using SHEsis software [46, 47]. LD between SNP pairs was evaluated utilizing the absolute value of Lewontin’s D′ and also Pearson’s correlation (r). To evaluate power of the study, we used Quanto (version 1.1) with parameters set as: a dominant model, a population risk of malaria of 2%, a minor allele frequency of 34% (the frequency for all 200 of our controls) for OR = 1.80 at 2-sides p value 0.05 [48, 49]. ANOVA and Mann-Whitney U test were employed for comparison of the DNA methylation levels between groups and along with Kruskal-Wallis method to check the effect of different genotypes and haplotypes on the methylation status.

## Results

### Western blotting analysis

**Fig 2:**
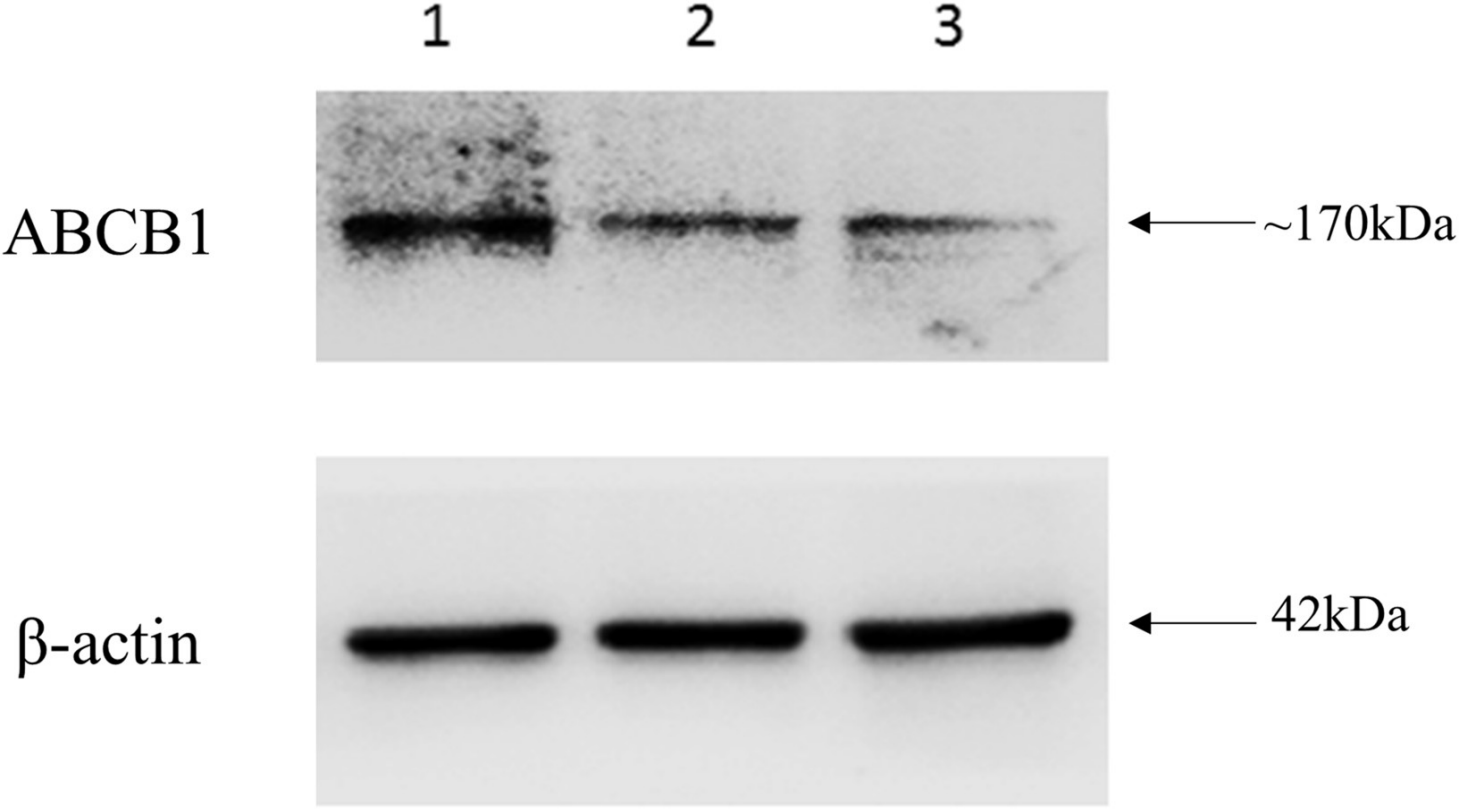
Western blotting analysis of anti-ABCB1 antibody and β-actin was used as internal control. (Lane 1: Individual with complicated malaria; Lane 2: Individual with un-complicated malaria; Lane 3: Healthy individual).

ABCB1 expression was measured and compared in all the three groups. ABCB1 protein levels were high in the leukocytes of the individual with complicated malaria when compared to uncomplicated malaria and control as shown in Fig 2. Densitometric analysis, normalized to β-actin loading controls, indicated nearly a threefold increase in ABCB1 protein expression in individual with complicated malaria.

### *ABCB1* SNPs association to malaria

**Fig 3:**
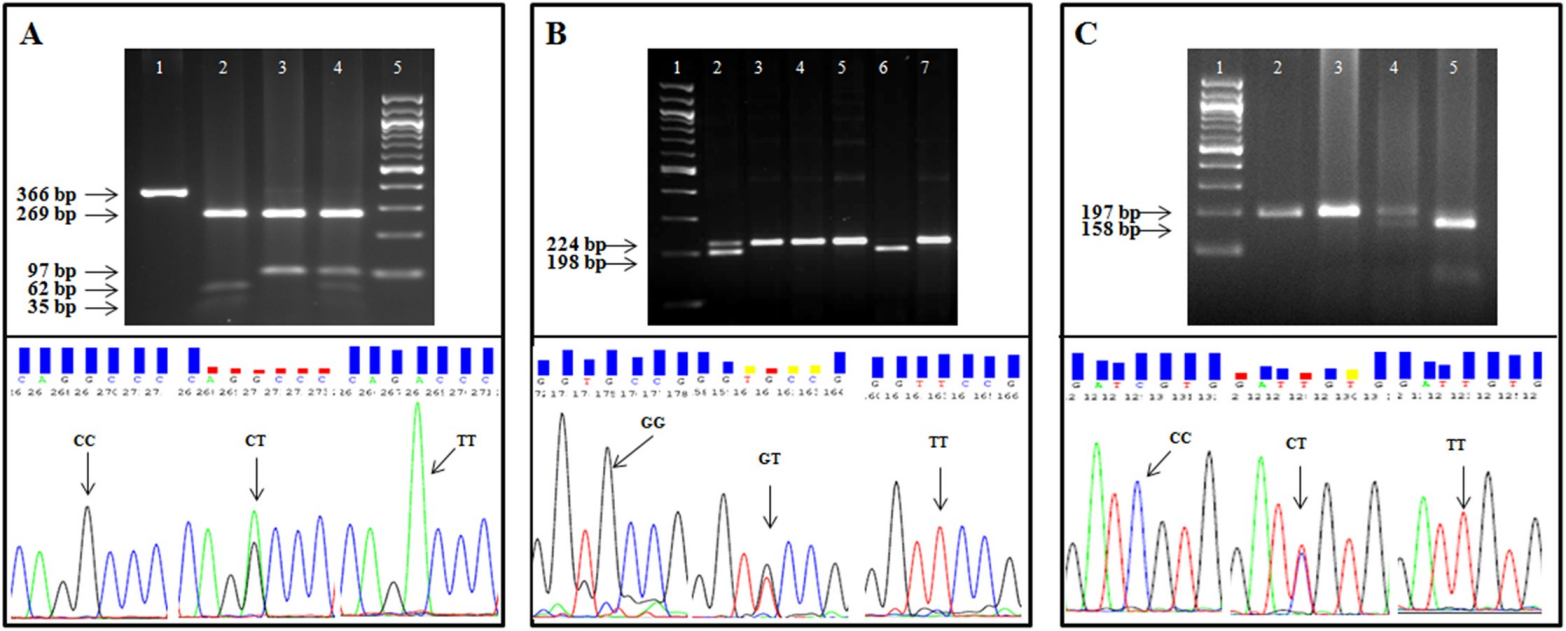
**Figure shows PCR-RFLP (upper half) and confirmatory DNA sequencing (lower half) experiments, for the three SNPs [rs1128503 (panel A), rs2032582 (panel B) and rs1045642 (panel C)] of *ABCB1* gene.** Panel A shows agarose gel electrophoresis of PCR amplicons (size, 366 bp) containing rs1128503 C>T polymorphism, digested with restriction enzyme HaeIII showing homozygous wild type CC individuals (lane 2) homozygous mutant type TT alleles (lane 3) and heterozygous CT variant (lane 4). The undigested PCR amplicon is shown in lane 1 and the DNA size marker is shown in lane 5. Panel B shows PCR-RFLP with BanI restriction enzyme for genotyping rs2032582G>T polymorphism. The wild type allele G containing PCR amplicon (of size, 224 bp) is digested by BanI to generate two fragments of 198 and 26 base pairs. The three genotypes corresponding to the fragment lengths, GG (lane 6), GT (lane 2) and TT (lane 3, 4, 5 and 7) are shown in the picture. Panel C shows agarose gel electrophoresis of PCR amplicons (size 197 bp) containing rs1045642 C>T polymorphism, digested with restriction enzyme Sau3A1 showing homozygous wild type CC individuals (lane 5) homozygous mutant type TT alleles (lane 2, 3) and heterozygous CT variant (lane 4). Here, the PCR amplicons with wild type C allele is digested into two fragments of size 158 bp and 39 bp whereas the mutant T allele containing fragment remain undigested. DNA sequencing results for each polymorphism are presented in the bottom half of the panels in the figure as the DNA sequences of wild type homozygotes, heterozygotes and mutant homozygotes of the three polymorphisms represented in the respective panels as indicated by arrows.

The allele and genotype frequencies of three *ABCB1* polymorphisms (rs1128503, rs2032582 and rs1045642) were tested to estimate the levels of association of the *ABCB1* gene SNPs with malaria caused by *Pf* are presented in Table 2. All the three SNP loci in all three malaria and control group were in HWE except rs1128503 (*p* = 0.02) and rs2032582 (*p* = 0.006) in uncomplicated malaria group. We determined that our study had 80% power to detect an association. The frequencies of the mutant allele (T) and genotype (TT) of rs2032582 polymorphism showed a significantly higher occurrence in complicated [T (OR = 1.9; *p* = 0.0009); TT (OR = 3.1; *p* = 0.006)] and in all malaria [T (OR = 1.6; *p* = 0.003); TT (OR = 2.2; *p* = 0.01)] groups indicating a higher risk for malarial disease than controls. The dominant model analysis of rs203582 SNP revealed an increased risk of malaria susceptibility in complicated and all malaria groups as shown in Table 2. The genotype and allele frequencies at other SNP loci, i.e., rs1128503 and rs1045642, did not show any significant difference between the three malarial groups and normal controls (*p* >0.05) except rs1128503 showed significant association with malaria susceptibility with complicated and all malaria groups as presented in Table 2. The *p* values were corrected with Bonferroni corrections. The RFLP band patterns and DNA sequencing electropherogram are shown in Fig 3.

**Table 2:** Genotype/allele frequency data of SNPs of *ABCB1* gene and the results of the test for genetic association with case (uncomplicated, complicated and all malaria) and control, with measures of statistical significance.

**Gene/ SNP**	**Genotype/** **allele**	**Uncomplicated malaria (n = 100)**	**Complicated malaria (n = 100)**	**Malaria** **(n = 200)**	**Controls** **(n = 200)**	**Models**	**Uncomplicated v/s control**		**Complicated v/s control**		**Complicated v/s uncomplicated**		**All malaria v/s control**	
							OR	^a^*p value*	OR	^a^*p value*	OR	^a^*p value*	OR	^a^*p value*
**rs1128503**	CC	32	24	56	68									
	CT	43	49	92	100									
	TT	25	27	52	32	Additive	1.6 (0.8–3.2)	NS	2.4 (1.2–4.8)	**0.03**	1.4 (0.7–3.1)	NS	2.0 (1.1–3.4)	NS
	CT+TT	68	76	144	132	Dominant	1.1 (0.6–1.8)	NS	1.6 (0.9–2.8)	NS	1.5 (0.8–2.8)	NS	1.3 (0.8–2.0)	NS
	CC+CT	75	73	148	168	Recessive	0.6 (0.3–1.0)	NS	0.5 (0.3–0.9)	NS	0.9 (0.5–1.7)	NS	0.5 (0.3–0.9)	**0.03**
	CC+TT	57	51	108	100	Co-dominant	1.3 (0.8–2.1)	NS	1.0 (0.6–1.7)	NS	0.8 (0.4–1.4)	NS	1.1 (0.8–1.7)	NS
	C	107	97	204	236									
	T	93	103	196	164	Allele	1.2 (0.9–1.8)	NS	1.5 (1.1–2.1)	**0.03**	1.2 (0.8–1.8)	NS	1.3 (1.1–1.8)	NS
**rs2032582**	GG	41	26	67	93									
	GT	37	49	86	78									
	TT	22	25	47	29	Additive	1.7 (0.9–3.3)	NS	3.1 (1.5–6.1)	**0.006**	1.7 (0.8–3.8)	NS	2.2 (1.3–3.9)	**0.01**
	GT+TT	59	74	133	107	Dominant	1.3 (0.8–2.0)	NS	2.5 (1.5–4.2)	**0.002**	1.9 (1.1–3.6)	NS	1.7(1.1–2.5)	**0.03**
	GG+GT	78	75	153	171	Recessive	0.6 (0.3–1.1)	NS	0.5 (0.3–0.9)	NS	0.8 (0.4–1.6)	NS	0.5 (0.3–0.9)	NS
	GG+TT	63	51	114	122	Co-dominant	1.1 (0.7–1.8)	NS	0.7 (0.4–1.1)	NS	0.6 (0.3–1.1)	NS	0.8 (0.5–1.2)	NS
	G	119	101	220	264									
	T	81	99	180	136	Allele	1.3 (0.9–1.9)	NS	1.9 (1.3–2.7)	**0.0009**	1.4 (0.9–2.1)	NS	1.6 (1.2–2.1)	**0.003**
														
**rs1045642**	CC	7	2	9	14									
	CT	21	36	57	59									
	TT	72	62	134	127	Additive	1.1 (0.4–2.9)	NS	3.4 (0.7–15.5)	NS	3.0 (0.6–15)	NS	1.6 (0.6–3.9)	NS
	CT+TT	93	98	191	186	Dominant	1.0 (0.4–2.6)	NS	3.6 (0.8–16.5)	NS	3.6 (0.7–18.2)	NS	1.6 (0.7–3.8)	NS
	CC+CT	28	38	66	73	Recessive	0.7 (0.4–1.1)	NS	1.1 (0.6–1.7)	NS	1.5 (0.8–2.8)	NS	0.8 (0.6–1.2)	NS
	CC+TT	79	64	143	141	Co-dominant	1.6 (0.9–2.8)	NS	0.7 (0.5–1.2)	NS	0.5 (0.3–0.9)	NS	1.0 (0.7–1.6)	NS
	C	35	40	75	87									
	T	165	160	325	313	Allele	1.3 (0.8–2.0)	NS	1.1 (0.7–1.7)	NS	0.8 (0.5–1.4)	NS	1.2 (0.8–1.7)	NS

Note: Odds ratio (OR) for single SNPs were calculated using the approximation of Woolf for the 95% confidence interval.

^a^p value = Fisher’s exact test p value after Bonferroni correction < 0.05 (in bold) considered to be significant. NS = non-significant

### The prediction of haplotypes

The haplotype analysis was performed for three polymorphisms of *ABCB1* by using SHEsis. The results of the haplotype analysis are shown in Table 3. Out of the possible 8 haplotypes involving the three polymorphic loci, T-T-T mutant haplotype was found uniquely associated with complicated (OR = 2.14; *p*< 0.001), uncomplicated (OR = 1.47; *p* = 0.03) and all malaria (OR = 1.75; *p* = 0.0002) groups whereas C-G-T haplotype was significantly associated with control (*p*<0.05). The haplotype conferring resistance to malaria disease consisted of only wild type alleles at the first two loci, whereas T-T-T haplotype showed significantly high risk for malaria susceptibility consisted of mutant alleles at three loci.

**Table 3:** Predicted haplotypes of the SNPs of *ABCB1* gene, and statistical analysis of the association to complicated, uncomplicated and malaria patients versus controls.

	**Haplotype**	**Uncomplicated malaria**	**Complicated malaria**	**Malaria**	**Control**	**χ2—value**	***p*–value**^a^	**OR (95% CI)**
**Complicated v/s control**	C-G-C	-	33.58(0.168)	-	64.45(0.161)	0.007	NS	1.019 [0.644–1.612]
	C-G-T	-	53.68(0.268)	-	146.50(0.366)	6.902	**0.008**	0.605 [0.415–0.882]
	C-T-T	-	5.03(0.025)	-	17.95(0.045)	1.541	NS	0.534 [0.196–1.459]
	T-G-T	-	13.73(0.069)	-	43.73(0.109)	2.853	NS	0.583 [0.309–1.097]
	T-T-T	-	87.55(0.438)	-	104.82(0.262)	17.458	**<0.001**	2.148 [1.496–3.084]
**Uncomplicated v/s control**	C-G-C	34.98(0.175)	-	-	64.45(0.161)	0.016	NS	1.029 [0.655–1.619]
	C-G-T	63.38(0.317)	-	-	146.50(0.366)	2.862	NS	0.732 [0.509–1.051]
	C-T-T	8.62(0.043)	-	-	17.95(0.045)	0.059	NS	0.902 [0.393–2.070]
	T-G-T	20.64(0.103)	-	-	43.73(0.109)	0.212	NS	0.878 [0.505–1.528]
	T-T-T	72.36(0.362)	-	-	104.82(0.262)	4.353	**0.037**	1.475 [1.023–2.127]
**Complicated v/s uncomplicated**	C-G-C	34.98(0.175)	33.58(0.168)	-	-	0.001	NS	0.990 [0.588–1.667]
	C-G-T	63.38(0.317)	53.68(0.268)	-	-	0.740	NS	0.827 [0.536–1.275]
	C-T-T	8.62(0.043)	5.03(0.025)	-	-	0.859	NS	0.593 [0.194–1.813]
	T-G-T	20.64(0.103)	13.73(0.069)	-	-	1.284	NS	0.664 [0.325–1.354]
	T-T-T	72.36(0.362)	87.55(0.438)	-	-	3.334	NS	1.456 [0.972–2.181]
**Malaria v/s control**	C-G-C	-	-	73.13(0.183)	64.45(0.161)	0.222	NS	1.093 [0.756–1.581]
	C-G-T	-	-	112.76(0.282)	146.50(0.366)	9.583	**0.001**	0.623 [0.461–0.841]
	C-T-T	-	-	17.76(0.044)	17.95(0.045)	0.038	NS	0.935 [0.478–1.831]
	T-G-T	-	-	34.11(0.085)	43.73(0.109)	1.957	NS	0.715 [0.446–1.146]
	T-T-T	-	-	160.38(0.401)	104.82(0.262)	13.482	**0.0002**	1.755 [1.298–2.372]

*^a^ p* value < *0.05 considered to be significant.*

NS = non-significant

### LD structure

Further, linkage disequilibrium for *ABCB1* gene was determined by using the SHEsis program. LD was calculated among SNP pairs using the D′ and r^2^ values between the 3 SNPs. The analysis showed strong linkage disequilibrium (D′ = 1 or > 0.75) between rs1128503, rs2032582 and rs1045642 polymorphisms in all three groups of malarial samples. This linkage block was found completely disrupted in the control samples due to the absence of significant LD between the three SNPs rs1128503, rs2032582 and rs1045642 (D′ < 0.6).

### DNA methylation analysis

**Fig 4:**
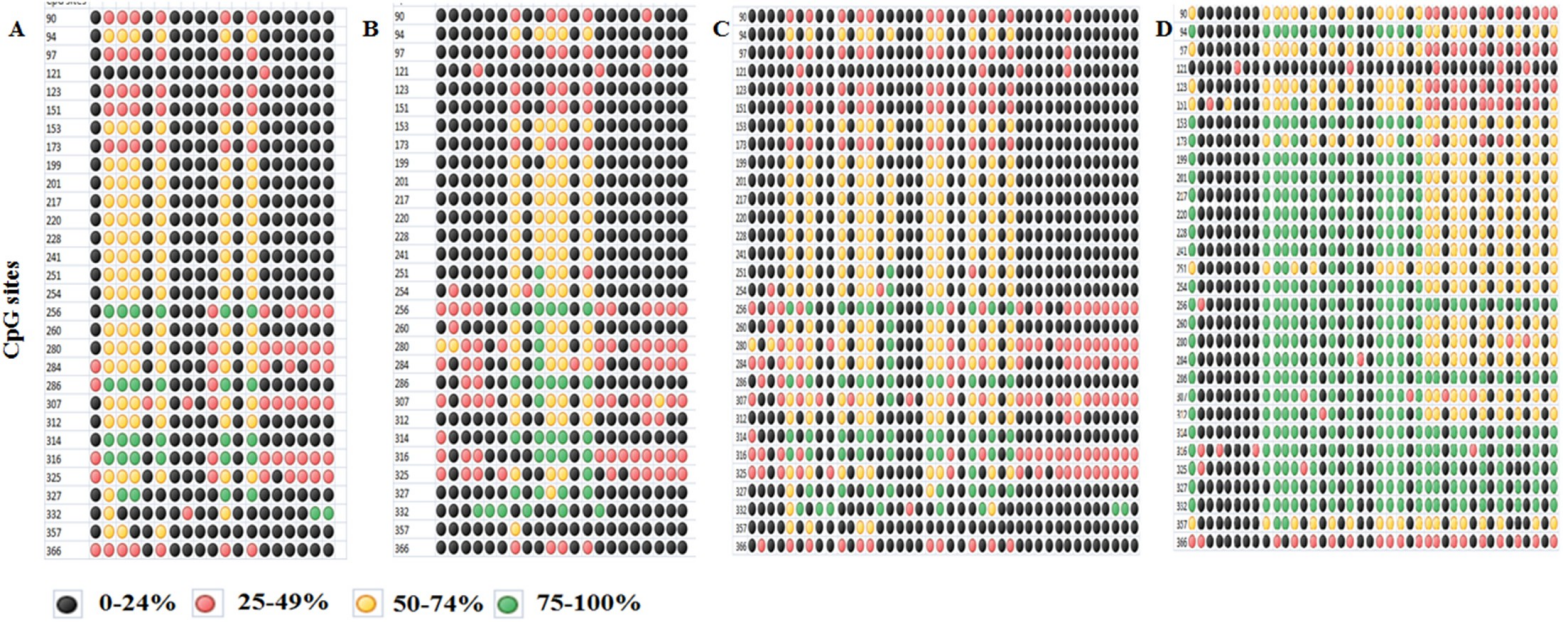
Dot plot of ABCB1 promoter DNA methylation at 30 CpG sites. Complicated malaria (Panel A), Uncomplicated malaria (Panel B), All malaria cases (Panel C) and Controls (Panel D).

Analysis of the sequenced samples showed an average of 21.2, 23.73, 22.41 and 42.35 percent DNA methylation of most of the CpG sites among the 30 CpG sites in *ABCB1* PCR product of all the samples in complicated, uncomplicated, all malaria and control (in both age-sex matched samples) groups respectively. Comparative analysis was done for all the three groups of malaria and control groups with DNA methylation percent values by using One-way ANOVA test, and the result was found to be significant (*p*< 0.05) as shown in Table 4. The distributions of mean DNA methylation levels for particular CpG sites in each sample are shown in Fig 4. DNA methylation analysis of *NNAT* gene promoter in randomly picked 20 samples from malaria and control groups revealed no significant difference in DNA methylation levels (data not shown).

**Table 4:** Results obtained from One-way ANOVA test of DNA methylation levels of *ABCB1* promoter in complicated, uncomplicated, all malaria and control groups.

Bonferroni's Multiple Comparison Test	t	^a^*p* value
Complicated Malaria vs Uncomplicated Malaria	0.5157	NS
Complicated Malaria vs All Malaria	0.2726	NS
Complicated Malaria vs Control	4.809	**0.001**
Uncomplicated Malaria vs All Malaria	0.2924	NS
Uncomplicated Malaria vs Control	4.110	**0.001**
All Malaria vs Control	4.999	**0.001**

^*a*^*p* value *= p value after Bonferroni correction < 0*.*05 (in bold) considered to be significant*. *NS = Non-significant*.

### Genotypes and DNA methylation of *ABCB1* correlation

Kruskal-Wallis test was performed to estimate the effect of genotypes of three polymorphisms on DNA methylation status of *ABCB1* gene both in all malaria case (n = 40) and control (n = 40) groups. Our study revealed a significant correlation of rs1128503 (*p* = 0.02) and rs2032582 (*p* = 0.02) polymorphisms with DNA methylation status in malaria cases. However, the 3 SNP loci did not show any significant correlation with DNA methylation status in control samples as shown in Table 5. Further, Mann-Whitney U test was employed between DNA methylation status of individuals with these genotypes wild v/s hetro, wild v/s mutant and hetro v/s mutant of rs1128503 and rs2032582 in all malaria cases. In malaria group, CC v/s TT and CT v/s TT of rs1128503 polymorphism and GG v/s TT and GT v/s TT of rs2032582 genotype individuals DNA methylation status were found significant different *p*< 0.05 in all malaria group.

**Table 5:** Correlation analysis of the three SNPs of *ABCB1* gene and DNA methylation levels in all malaria cases and controls.

	Genotype	Subjects	Mean Methylation (%)	Range Methylation (%)	*p*–value^a^
All malaria cases					
rs1128503	CC	19	22.40	5.2–59.7	**0.02**
	CT	16	27.32	6.8–57.8	
	TT	5	6.66	2.4–11.2	
rs2032582	GG	18	26.06	5.2–59.7	**0.02**
	GT	17	23.16	5.9–57.4	
	TT	5	6.66	2.4–11.2	
rs1045642	CC	7	31.26	9.0–59.7	**0.22**
	CT	12	21.63	6.8–57.3	
	TT	21	19.89	2.4–57.8	
Controls					
rs1128503	CC	19	41.5386	3.3–85.0	0.23
	CT	15	36.82	4.0–85.5	
	TT	6	58.77222	1.9–86.7	
rs2032582	GG	27	43.06543	3.3–85.5	0.06
	GT	7	24.87143	1.9–84.5	
	TT	6	59.55	6.6–86.7	
rs1045642	CC	6	54.80556	6.6–85.5	0.48
	CT	14	43.43333	4.5–84.1	
	TT	20	37.86333	1.9–86.7	

^*a*^ Kruskal-Wallis *p* value *< 0*.*05 considered to be significant*.

### Haplotypes and DNA methylation of *ABCB1* correlation

DNA Methylation status of each haplotype was different. To evaluate the effect of haplotypes on DNA methylation status only C-G-T and T-T-T haplotypes were considered, which were found significant in the study. All the two haplotypes were divided into two groups of carriers (individual with the haplotype) and non-carriers (individual without the haplotype) in both all malaria case (n = 40) and control (n = 40) groups. The Mann-Whitney U test was applied to two groups of each haplotype in all malaria cases and controls. No significant difference *p*> 0.05 was observed for C-G-T and T-T-T haplotypes in both the groups.

### Global DNA methylation analysis

**Fig 5:**
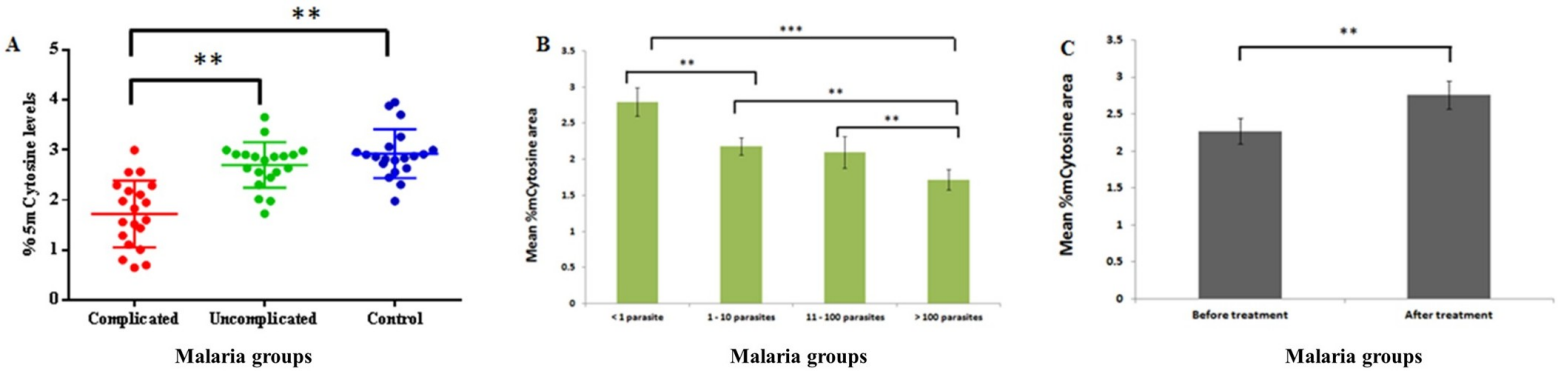
Global methylation estimation by RP-HPLC. A. Global methylation values (5mC) in malaria patients (with and without complications) and controls. Each dot represents the total methylation cytosine content of the individual sample. B. Global methylation values (5mC) in malaria patients (with different parasite levels). C. Global methylation values (5mC) in malaria patients (before and after treatment). Patient blood was collected twice at the time of admission (before medication) and after 7days of treatment. Within the groups mean ± standard error was shown in lines. The aster sign indicates the p value significance. **P<0.01, *P<0.05.

Global methylation analysis was performed on age and gender matched participants in malaria subjects (n = 40) and controls (n = 20). Malaria subjects were categories into different groups such as complicated (n = 20) and uncomplicated (n = 20) malaria and individuals with different levels of parasite load (as per QBC analysis) in the blood. Global methylation levels were also evaluated in malaria patients before (n = 10) and after treatment (n = 10) of malaria infection irrespective of complications. We observed a significantly less global methyl cytosine content (*p*<0.05) in the group of individuals with high parasitemia, complications and before treatment when compared to low parasitemia, non-complications, after treatment and controls individuals as shown in Fig 5.

### *ABCB1* DNA promoter activity

**Fig 6:**
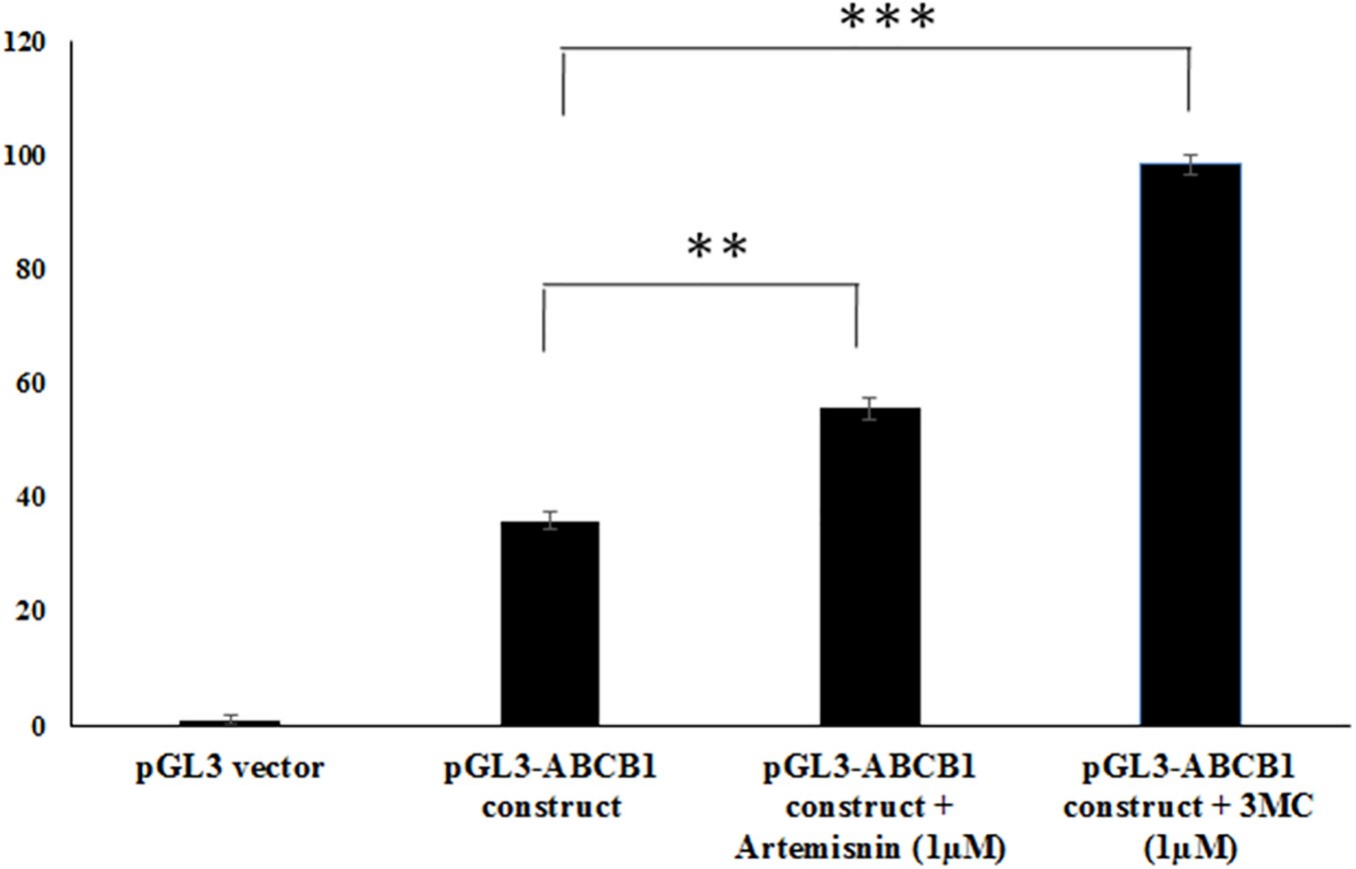
Promoter activity analysis of ABCB1 constructs using luciferase assays. Showing pGL3- ABCB1 construct (contains 949bp DNA fragment) promoter activity results upon artemisinin and 3-methylcholanthrene treatments.

To test various pGL3 and ptk-luc constructs consisting of *ABCB1* promoter sequences for activity, these were transiently transfected in HepG2 cells and subsequently testing for luciferase activity. The test ptk-luc-ABCB1 construct demonstrated 17% higher promoter activity compared to pGL3-basic vector as well as empty ptk-luc vector. Promoter activity of pGL3-ABCB1 construct was 35.9% higher when compared to pGL3-basic vector. Promoter activity of pGL3-ABCB1 construct showed significantly higher after treatment with 1μM artemisinin (55.6% higher) and 1μM 3MC (98.3%) when compared to pGL3-basic vector as shown in Fig 6.

## Discussion

With nearly half of the world population at risk for malaria, only a small proportion (1–3%) of *Plasmodium* infection leads to severe cases while other remains asymptomatic or remain as uncomplicated malaria [50]. In order to identify the risk of individual developing malaria and to predict the outcome, we analysed multiple alterations in *ABCB1* and related its impact on the disease. Genetic variants, their functional relevance to *Pf* as well as to other infectious diseases [51] can provide important information on the aetiology of the disease. Previously described protective alleles against malaria relate to genes that are involved with the host immune response such as cytokines, endothelial receptor, complement regulatory gene, *HLA* gene and RBC structure [50–54]. These polymorphisms in immune response genes do not cause genetic pathology in the host by themselves, but rather are associated with malaria severity [53]. P-gp as coded by *ABCB1* gene is involved in the transfer of various substances out of cells, including anticancer drugs, immunosuppressants, HIV protease inhibitors, cardiac drugs and adenoreceptor antagonists [55]. Polymorphisms in *ABCB1* gene have been reported to potentially alter these transport functions thereby modulating the disease susceptibility and drug response in various disease conditions [19]. Our western blot analysis of ABCB1 protein in peripheral mononuclear cells showed higher expression in individuals with malaria than the control group, suggesting that it may have an important role to play in the aetiology of the disease.

There have been several attempts to correlate polymorphisms, expressions and mRNA stability of *ABCB1* in human diseases with limited success and sometimes even the opposite effect [56]. In our study, the genetic association of rs1128503 and rs2012582 *ABCB1* polymorphisms between malaria and control subjects were significantly different as shown in Table 2. The G2677A/T non-synonymous SNP situated at exon 21 alters drug transport by affecting induced ATPase activity [57]. The previous study has reported that the individual with homozygous T allele of 1236C>T as well as 2677G>T polymorphisms have high clearance of imatinib that was used to treat chronic myeloid leukemia or gastrointestinal stromal tumors [58]. Similar results were observed in the study, wherein, the population homozygous for T allele of rs1128503 requires the higher dose of methadone than individuals homozygous for wild type alleles [59]. Higher expression of ABCB1 protein in complicated malarial group was observed in our study, and from other diseases suggest that these polymorphisms (rs1128503 and rs2032582) may be associated with over expression of P-gp that may lead to resistance to anti-malarial drugs and hence requires increased doses of artemisinin for effective treatment. The 3435C>T SNP was found to be associated with decreased intestinal P-gp expression and hence increased digoxin bioavailability [60] and negative correlation of rs1045642 to malaria in our study supports the similar finding of reduced P-gp expression.

In order to obtain a discernible association with malaria arising from linkage disequilibrium between typed SNP and a primarily associated polymorphism [50], we performed haplotype prediction with SHEsis and found that haplotype T-T-T to be associated with malaria. This indicates that thepresence of mutant T alleles increases the risk to the disease significantly and C-G-T was found to associate primarily with controls uniquely conferring resistance to malaria with all wild alleles at the first two loci (Table 3). This was in accordance with the study performed by Levran *et al*. (2008) who found that individual bearing mutant genotype pattern at rs1045642, rs2032582 and rs1128503 SNPs of *ABCB1* gene have a chance of requiring 5 fold higher methadone dose whereas individuals with heterozygous for these above mentioned SNPs have approximately 3 fold chance of stabilizing at lower methadone dose [59].

Many attempts have been made to deduce the relationship between polymorphisms in host genetic factors that is responsible for the observed variation towards susceptibility to malaria; however, this has failed to establish the conclusive association [61]. Modiano *et al* (2001) have studied the known or suspected genetic factors of resistance (such as HLAB*5301, HbS, G6PDA^-^) to malaria with an aim to ascertain their role in lower susceptibility to the disease among 3 sympatric ethnic groups–Fulani, Mossi and Rimaibe. They observed that these resistance genes even though present at high frequency among all 3 ethnic groups, show interethnic heterogeneities towards the susceptibility to malaria [62]. Similarly, some of the SNPs show a high correlation to malaria whereas, the same SNP shows no association when the study is carried out either in other population, geographical area, endemic/non-endemic regions, ethnic groups or tissue samples [51, 57, 63]. There are SNPs reported which even show the inverse correlation with respect to sex and age [64]. Thus there must be other non-genetic factor such as epigenetic factors (that varies in people of different ethnic group, age, sex, geographical area etc.) that may impact susceptibility or resistance to diseases. It has been found that human genetic factors contribute to about 25% of the variation observed [50]

DNA methylation of *ABCB1* gene has been reported to regulate drug response. For example, hypomethylation of the *ABCB1* downstream promoter region was found to be significantly associated with drug resistances in breast tumor cells due to increased expression of *ABCB1* transcripts [65, 66]. In our study, we show that the downstream promoter (+245 to +679) of *ABCB1* has low levels of DNA methylation in complicated, uncomplicated and combined malaria groups when we compared to controls. Over expression of the *ABCB1* gene can lead to poor treatment response in the diseases. Previous studies have demonstrated that in cell lines with higher DNA methylation of the *ABCB1* promoter correlated with lack of expression [67, 68]. *In vitro* studies which manifest increase in promoter activity of pGL3-ABCB1 constructs upon treatment of 1μM of artemisinin may represent *in vivo* situation of global DNA methylation patterns as observed in malarial subjects who have undergone treatment when compared to untreated controls. It is indeed possible that artemisinin itself may provide a feedback mechanism to control ABCB1 activity. Previously it has been reported that artemisinin and parthenolide are capable of initiating over expression of *ABCB1* gene through xenosensing nuclear receptors by activating pregnane X and constitutive androstane receptors [36].

Correlation analysis of genotypes and haplotypes with DNA methylation status revealed the significant association between the genotypes and DNA methylation only. Global methylation analysis showed reduced 5-mC levels in DNA of individuals from the malaria group as compared to the control group. This change in 5mC levels may be due to a) parasite and its by-products may change methylation levels of the host genome for its survival and b) host immune system may respond as defence mechanism upon parasite invasion to clear the parasite or to reduce the severity of the disease. Global methylation analysis showed that malarial infection induces alterations in global 5-mC levels in the host DNA. A significant difference in global 5-mC between malarial and non-malarial participants suggest that DNA methylation differences could discriminate disease phenotypes.

Upon infection by *Pf*, several immunological responses are induced as a first line defence mechanism [69]. One of such response is the release of cytokines, including inflammatory cytokines such as TNF-α, IL-1 and IL-6 [29–31] as well as anti-inflammatory cytokines IL-4, IL-10, TGF-β [70]. Earlier studies have reported the elevated level of TNF-α, IL-6, NO, IL-1b, TGF-β, and IL-10 in patients with complicated *Pf* malaria [71, 72]. However, the pathogenesis in *Pf* malaria and its severity is determined by the balance between pro-inflammatory and anti-inflammatory cytokines [72]. Many of the regulatory pathways that are usually triggered by pro-inflammatory cytokines include nuclear factor kappa B (NF-κB), signal transducer and activator of transcription (STAT) and activator protein-1 (AP-1). Human *ABCB1* promoter contains *cis*-elements for NF-κB, AP-1 and STAT [26] and hence increased level of pro-inflammatory cytokines could be involved in the over-expression of ABCB1 protein.

*ABCB1* is driven by two different promoters and among which downstream promoter has been the major promoter frequently used for transcription in most cell types [73–76]. Our study demonstrated the GC-rich box within the downstream promoter is regulated by DNA methylation. We have shown that +245 to +679 promoter region of *ABCB1* that covers the downstream promoter region is significantly methylated in control samples as opposed to samples from malaria subjects. Previously, differential methylation in this region has been demonstrated in various other cell types [77–79]]. It is known that methylation of the promoter is one of the major mechanisms for control of gene expression and hypomethylation of downstream promoter region of *ABCB1* in malaria patient as demonstrated in our current study suggests that reduced methylation level upon infection allow trans-acting factors such as LRPPRC (leucine rich pentatricopeptide repeat multifunctional family) [80–82] and pro-inflammatory cytokines activated NF-κB, AP-1 and STAT to participate in the transcription of *ABCB1* gene [27].

Malaria parasite invasion into the erythrocytes leads to degradation of nearly 80% of hemoglobin. In fact, it has been estimated that each parasite infected RBC may cause the elimination of approximately ten healthy RBCs and these lead to an increase in the amount of hemoglobin degraded product such as bilirubin and heme [83]. *Plasmodium* species convert heme to hemozoin as a mechanism to overcome the toxic effect of heme as they lack heme oxygenase (HO-1) enzyme and direct or indirect effect of hemoglobin degraded byproduct on hemozoin shows the deleterious effect on parasite [84]. Because of lipophilic nature, heme and its derivative hemin intercalate in the membrane bilayer and oxidizes membrane phospholipids. This may lead to disturbance in the integrity of RBC membranes and in turn, heme may hinder parasite penetration in the RBC [85]. It has been demonstrated that hemin promotes exposure of phosphatidylserine in the outer surface of RBC signaling for its clearance by the macrophage. However, hemin also shows the toxic effect to macrophages [86].

The ability of heme to complex with serum albumin [84] disturbs the normal transport pathway of unconjugated bilirubin (UCB) to the liver for conjugation and further excretion from the body. Bilirubin levels are usually increased in malarial patients [87]. UCB, although has a cyto-protective role, the cytotoxic effect of UCB to RBCs, peripheral blood mononuclear cells, immune cells and to neuronal and non-neuronal tissue has been observed [84, 88–91]. A study by Kaufmann *et al*., showed enhanced binding of UCB to blood erythrocyte when the UCB/albumin molar ratios exceed 1.0, which is the immediate target for unconjugated bilirubin [92] leading to ultimate morphological changes, cell lysis, and loss of membrane lipids [93]. At the same time, bilirubin is also known to inhibit hemozoin formation thereby interfering with the survival of the malarial parasites [84, 94]. Hence, the deleterious effect of the increase in hemoglobin degraded byproducts-heme and bilirubin on both host and parasite may require a mechanism to overcome the effect. Over expression of Pgp, which is the efflux protein, could be one mechanism by which macrophages, as well as parasite, avoid the toxic effect of heme and bilirubin. Bilirubin is the substrate for Pgp [95, 96] and hence efflux of bilirubin by ABCB1 protein could benefit parasite as well as the host cells. Therefore, it is conceivable that higher expression of ABCB1 in lymphocytes of *Pf* subjects is due to the enhanced cytokine expression and that it acts to promote efflux of compounds such as bilirubin or antimalarial drug to facilitate its survival.

In conclusion, we have made an attempt to decipher the multiple mechanisms that control *ABCB1* gene during malaria infection and demonstrate that both genetic and epigenetic mechanisms as in separable events may significantly contribute to malarial parasite infection, host-response towards inflammation, toxic by-products and drug response, and may participate in the aetiology of the disease.

## Supporting information

S1 Table The inclusion and exclusion criteria of study participants. (DOCX)
