# Supplementary material for: Genetic and epigenetic changes in host ABCB1 influences malaria susceptibility to Plasmodium falciparum
Source: PLoS One. 2017 Apr 19;12(4):e0175702. doi: 10.1371/journal.pone.0175702 (PMC5397027; doi:10.1371/journal.pone.0175702)
Supplement: S1 Table — (DOCX) [file pone.0175702.s001.docx]

**S1 Table.** **The inclusion and exclusion criteria of study participants**

|  | Inclusion criteria | Exclusion criteria |
| --- | --- | --- |
| Recruitment of individual with malaria infection | Age between 5 to 65 | Individuals less than 5 years old and more than 65 years old |
|  | Individuals from same malaria endemic area (Dakshin Kannada) | Individuals not from same area |
|  | Individuals with *P. falciparum* infection only | Individuals with mixed infection of *P. falciparum* and *P. vivax* |
|  | Individuals of any sex (male or female) | Individuals with *P. falciparum infection* but also have any other disease |
|  | Individuals willing to give signed informed consent | Individuals not willing to give signed informed consent |
| Recruitment of healthy control participant | Age between 5 to 65 | Individuals less than 5 years old and more than 65 years old |
|  | Individual from Dakshin Kannada | Individuals not from Dakshin Kannada |
|  | Healthy individual with no history of malaria and no other infection at the time of sample collection | Healthy individual with no past history of malaria but with other infection during the time of sample collection |
|  | Individuals of any sex (male or female) |  |
|  | Individuals willing to provide signed informed consent | Individuals not willing to give signed informed consent |
